# Supplementary material for: MetaRibo-Seq measures translation in microbiomes
Source: Nat Commun. 2020 Jun 29;11:3268. doi: 10.1038/s41467-020-17081-z (PMC7324362; doi:10.1038/s41467-020-17081-z)
Supplement: Supplementary file 10 — Supplementary Data 7 [file 41467_2020_17081_MOESM10_ESM.zip › File2/Confidence_High_Taxonomy/441281_out.krona.html]

Javascript must be enabled to view this page.

members
magnitude
magnitudeUnassigned
count
unassigned
taxon
rank

441281\_out

57

2
superkingdom
57

1239
57
phylum

57
class
186801

186802
57
order

541000
52
family

1263
5

SRS046712\_contig\_number\_contig-100\_252.253SRS053214\_contig\_number\_18461SRS098571\_contig\_number\_51998SRS1041033\_contig\_number\_13710SRS148721\_contig\_number\_contig-100\_3594.270968
52
genus

species

SRS015431\_contig\_number\_35706SRS018351\_contig\_number\_16813SRS052027\_contig\_number\_16298SRS147022\_contig\_number\_13470
4
2293176

1
species

SRS049712\_contig\_number\_contig-100\_255.130029
1095771

2293224

SRS017433\_contig\_number\_1747SRS020869\_contig\_number\_34382SRS049402\_contig\_number\_17461SRS104036\_contig\_number\_6545SRS104485\_contig\_number\_5198SRS142712\_contig\_number\_321SRS148253\_contig\_number\_8128
species
7

2293232
4
species

SRS017521\_contig\_number\_45949SRS019068\_contig\_number\_85056SRS058070\_contig\_number\_26885SRS105153\_contig\_number\_31684

2293208
4

SRS018623\_contig\_number\_14489SRS077552\_contig\_number\_18356SRS143991\_contig\_number\_10392SRS144362\_contig\_number\_32775
species

4

SRS017916\_contig\_number\_18369SRS018427\_contig\_number\_28985SRS1041138\_contig\_number\_7079SRS148159\_contig\_number\_41542
species
2293183

species

SRS142503\_contig\_number\_59217
1
2293207

2292069
1

SRS077502\_contig\_number\_contig-100\_901.108497
species

2293198
5
species

SRS018656\_contig\_number\_contig-100\_2361.64904SRS018836\_contig\_number\_13395SRS050422\_contig\_number\_contig-100\_1782.183295SRS144183\_contig\_number\_33899SRS147346\_contig\_number\_22817

1

SRS020233\_contig\_number\_14503
species
1897020

species

SRS014313\_contig\_number\_contig-100\_2042.49258SRS023526\_contig\_number\_9550SRS043701\_contig\_number\_7699SRS1041091\_contig\_number\_contig-100\_1252.126279SRS143342\_contig\_number\_15658SRS148511\_contig\_number\_7566SRS150029\_contig\_number\_17703SRS893259\_contig\_number\_124SRS893270\_contig\_number\_contig-100\_2069.116078
9
2293241

2302958
species

SRS149879\_contig\_number\_27410
1

2293158
3

SRS017191\_contig\_number\_5954SRS043411\_contig\_number\_15084SRS055982\_contig\_number\_contig-100\_174.100741
species

2293192
species

SRS015782\_contig\_number\_41330SRS104311\_contig\_number\_45958
2


SRS971275\_contig\_number\_21305
5
186803
1
family

841
genus
1

species

SRS023914\_contig\_number\_19424
1
512314

572511
3
genus

2

SRS050941\_contig\_number\_19431SRS100021\_contig\_number\_7205
species
40520

1
species

SRS050752\_contig\_number\_27306
418240
